# Tomographic aspects of penetrating thoracic trauma: injuries from firearms and other weapons

**DOI:** 10.1590/0100-3984.2016.0167

**Published:** 2017

**Authors:** Alessandro Severo Alves de Melo, Luiza Beatriz Melo Moreira, Fernanda Miraldi Clemente Pessoa, Nara Saint-Martin, Roger Ancilotti Filho, Arthur Soares Souza Jr., Edson Marchiori

**Affiliations:** 1 PhD, MD, Radiologist, Adjunct Professor at the Universidade Federal Fluminense (UFF), Niterói, RJ, Brazil.; 2 MD, Radiologist at the Hospital Universitário Antônio Pedro da Universidade Federal Fluminense (HUAP-UFF), Niterói, RJ, Brazil.; 3 MD, Resident at Hospital Universitário Antônio Pedro da Universidade Federal Fluminense (HUAP-UFF), Niterói, RJ, Brazil.; 4 PhD, MD, Radiologist, Faculdade de Medicina de São José do Rio Preto (Famerp) and Ultra X, São José do Rio Preto, SP, Brazil.; 5 Full Professor at the Universidade Federal do Rio de Janeiro (UFRJ), Rio de Janeiro, RJ, Brazil.

**Keywords:** Thoracic injuries, Wounds, penetrating, Tomography, X-ray computed, Traumatismos torácicos, Ferimentos penetrantes, Tomografia computadorizada

## Abstract

**Objective:**

The aim of this study was to analyze the various computed tomography findings in penetrating chest trauma, as well as to determine the frequency and extent of the lesions.

**Material and Methods:**

We studied the computed tomography findings from 40 cases of penetrating thoracic trauma, of which 35 (85.8%) were gunshot wounds and 5 (14.2%) were caused by another type of weapon.

**Results:**

Pulmonary lesions were found in 39 cases (97.5%), manifesting as contusions in 34 cases (85%), atelectasis in 8 (20%), lacerations in 1 (2.5%) and hematomas in 1 (2.5%). Hemothorax was seen in 31 cases (77.5%), and pneumothorax was seen in 22 cases (55%). Mediastinal lesions were observed in 8 cases (20%), including mediastinal hematoma in 3 cases (7.5%), hemopericardium in 3 (7.5%), and pneumomediastinum in 2 (5%). Diaphragmatic rupture was seen in 2 cases (5%).

**Conclusion:**

In patients with penetrating thoracic trauma, computed tomography of the chest is an important tool for characterizing the affected organs and evaluating the path of injury, as well as the severity and extent of the lesions. The images obtained are also useful in estimating the risk of death and determining the best therapeutic approach.

## Abstract (pt)

**Objetivo:**

Analisar os diferentes achados da tomografia computadorizada no trauma torácico aberto e determinar sua frequência e extensão.

**Materiais e Métodos:**

Neste trabalho foram estudados os aspectos tomográficos de 40 pacientes com trauma torácico aberto, sendo 35 casos causados por lesão por arma de fogo (85,8%) e 5 por arma branca (14,2%).

**Resultados:**

As lesões pulmonares predominaram, tendo sido observadas em 39 casos (97,5%), manifestadas sob a forma de contusões em 34 (85%), atelectasias em 8 (20%), lacerações em 1 (2,5%) e hematomas em 1 (2,5%). Hemotórax foi demonstrado em 31 casos (77,5%) e pneumotórax em 22 casos (55%). Lesões mediastínicas estiveram presentes em 8 casos (20%), com hematoma mediastínico em 3 casos (7,5%), hemopericárdio em 3 (7,5%) e pneumomediastino em 2 (5%). Lesão diafragmática foi observada em 2 pacientes (5%).

**Conclusão:**

A tomografia computadorizada de tórax, nos casos de trauma torácico aberto, permite caracterizar os órgãos acometidos, avaliar o trajeto das lesões, sua gravidade e extensão, sendo útil na estimativa de risco de vida e na escolha das abordagens terapêuticas.

## INTRODUCTION

Trauma is one of the leading causes of death and disability in the world today, especially in the younger population. Thoracic lesions represent a major aggravating factor in the evolution of patients with multisystem trauma, accounting for 20% of all deaths of traumatic origin^(1,2)^. Trauma is the third leading cause of death in Brazil, thoracic trauma accounting for one in four of such deaths. However, there have been no statistical studies specifically addressing thoracic trauma in Brazil^(1,2)^. In 2013, there were 151,000 accidental deaths in Brazil, corresponding to 75 deaths per 100,000 population^(1,2)^, which demonstrates the great relevance of the study of trauma. A study of trauma mortality in the city of São Paulo in 2011 showed that penetrating trauma was the predominant cause of traumatic injuries, accounting for 43% of the cases^(3)^.

Computed tomography (CT) represents a significant advance in the modern approach to trauma and in the immediate management of cases. CT is quite effective in evaluating traumatic lesions of the skull, face, spine, thorax, abdomen, and pelvis, allowing a more detailed study of the injuries of victims of trauma and therefore playing an essential role at trauma centers^(2)^. Chest CT presents greater sensitivity and specificity than does chest X-ray in the detection and evaluation of the extent of traumatic injury to the lung parenchyma, pleural space, mediastinum, and diaphragm^(4,5)^.

Trauma can induce various lesions in the intrathoracic organs. CT detects those lesions, potentially producing a broad array of findings. Pulmonary contusions are the most common parenchymal lesions, followed by atelectasis, lacerations, and hematomas. In the pleural space, hemothorax predominates, followed by pneumothorax. Other possible lesions include aortic injury, mediastinal hematoma, diaphragmatic injury, pneumomediastinum, soft tissue emphysema, and hemopericardium. Early diagnosis of some of these lesions can be critical for patient survival. The objectives of this study were to analyze the various CT findings in penetrating thoracic trauma, to calculate its frequency, and to determine its extent.

## MATERIALS AND METHODS

This was a retrospective descriptive study of 40 patients with penetrating thoracic trauma. Cases were selected consecutively from among those treated at a number of hospitals in the city of Rio de Janeiro. The study was evaluated and approved by the Research Ethics Committee of the Antônio Pedro University Hospital of the Fluminense Federal University. Patient ages ranged from 16 to 70 years, with a mean of 33 years. There was a predominance of males, who accounted for 82.4% of the cases (*n* = 34). Only 6 patients (17.6%) were female. The inclusion criteria were having undergone chest CT within the first 12 hours after the traumatic event and showing detectable intrathoracic traumatic lesions on the chest CT scan.

Because the chest CT scans were obtained at multiple institutions, they were performed in different helical tomography scanners, with various protocols. Images were acquired from the sternal foramen to the upper abdomen. CT scans were performed by two experienced thoracic radiologists, working independently, and discordant results were resolved by consensus. The chest CT images were analyzed in order to identify and characterize the pulmonary, pleural, mediastinal, and diaphragmatic lesions resulting from trauma caused by firearms or other weapons. Pulmonary lesions were evaluated in terms of their pattern and extent. Bruises, lacerations, hematomas, and atelectasis were also observed. Pulmonary contusion corresponds to a parenchymal lesion that manifests on CT as consolidation, ground-glass attenuation, or a mixture of the two. Pulmonary laceration was characterized on CT by the presence of air within a pulmonary contusion. Pulmonary hematoma represents a parenchymal lesion characterized by rounded opacities, always containing air or an air-fluid level. Pleural lesions were characterized in the presence of air (pneumothorax) and fluid of variable density (hemothorax) in the pleural space. The mediastinal lesions observed were pneumomediastinum, mediastinal hemorrhage, and hemopericardium. The diaphragmatic lesions were represented by herniation of the abdominal contents to the thorax, caused by rupture of the hemidiaphragm. The criteria used in defining these findings were those established in the Fleischner Society Glossary of Terms^(6)^. The terminology used is that presented in the terminology consensus of the Brazilian College of Radiology^(7)^ and the Imaging Committee of the Brazilian Thoracic Association^(8)^.

## RESULTS

In our sample, penetrating thoracic trauma was caused by gunshot in 35 cases (87.5%) and by injury from another type of weapon in 5 cases (12.5%). Penetrating thoracic trauma by firearm was accompanied by pulmonary contusion in 34 cases, hemothorax in 26, pneumothorax in 18, pulmonary atelectasis in 6, soft tissue emphysema in 3, hemopericardium in 3, hemomediastinum in 3, diaphragmatic lesion in 2, and pneumomediastinum in 2. Penetrating thoracic trauma caused by injury from other weapons resulted in pulmonary contusion, hemothorax, and pneumothorax in all 5 cases. Rib fractures were observed in 12 cases (30%). The main tomographic findings were as follows: pulmonary contusion, in 39 cases; hemothorax, in 31; pneumothorax, in 22; atelectasis, in 8; mediastinal hematoma, in 3; hemopericardium, in 3; pneumomediastinum, in 2; diaphragmatic injury, in 2; pulmonary laceration, in 1; and pulmonary hematoma, in 1. The organs most often affected by the penetrating trauma were the lungs, which were injured in 39 cases (97.5%). Pleural lesions were identified in 34 cases (85%). Mediastinal and diaphragmatic lesions were present in 8 (20%) and in 2 (5%) of the cases, respectively. Metallic foreign bodies were found in 10 cases (25%): metal fragments of a firearm projectile in 9 cases and a sharp object (knife) in 1 case. Pulmonary contusions were observed in all 10 of those cases: hemothorax was observed in 8, pneumothorax was observed in 7, and hemomediastinum was observed in 1.

### Lung parenchyma

**Figure 1:**
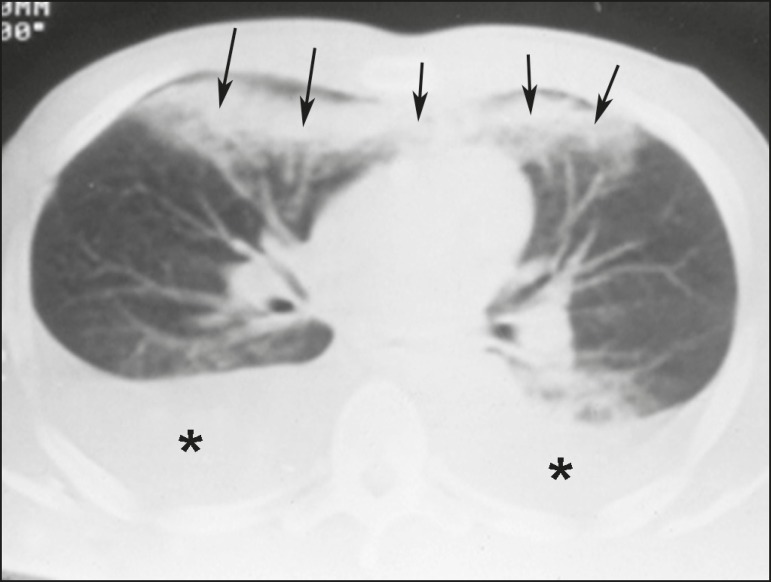
A 26-year-old male gunshot victim. Axial CT scan of the chest demonstrating tubular consolidation affecting the middle lung lobe and lingula, representing pulmonary contusion along the path of a firearm projectile (arrows). Note the bilateral pleural effusion (asterisks).

**Figure 2:**
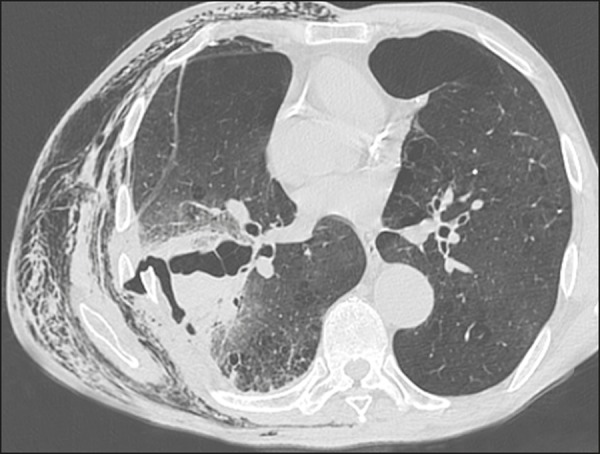
A 65-year-old male victim of a knife attack. Axial CT scan of the chest showing laceration and contusion of the right lung, accompanied by a misaligned rib fracture and lesion of the adjacent intercostal musculature, with hemopneumothorax. Extensive soft-tissue emphysema related to the ipsilateral chest wall. Left anterior pneumothorax can also be seen.

**Figure 3:**
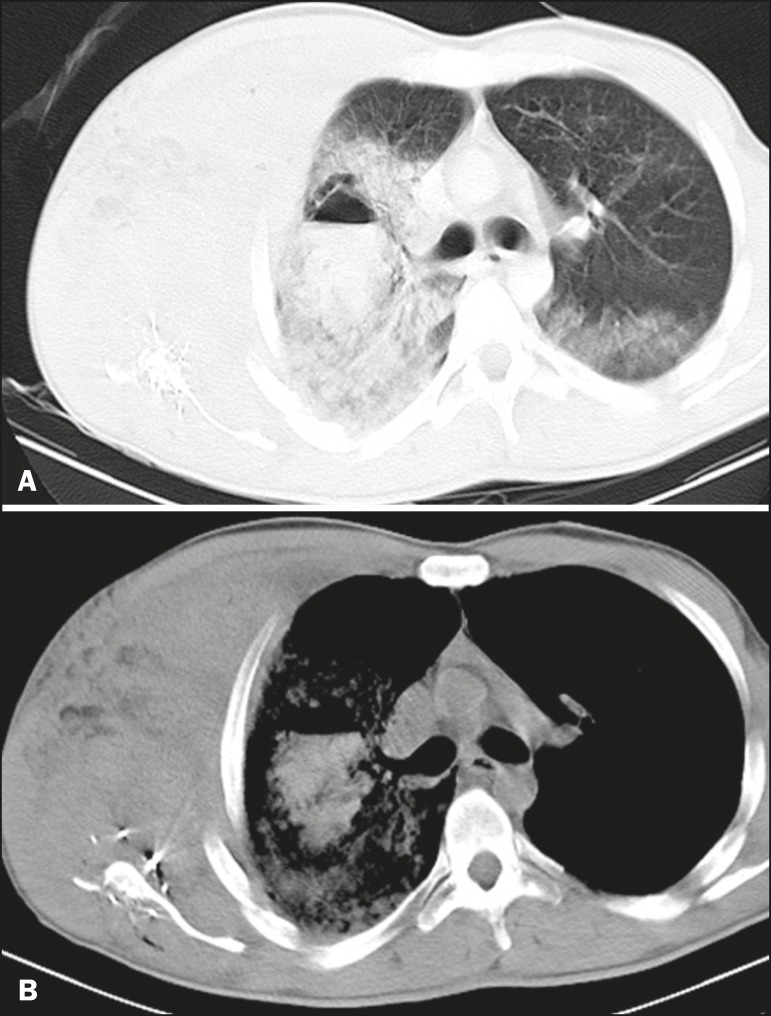
A 35-year-old male gunshot victim. Axial CT scans of the chest with a lung window (A) and a mediastinal window (B) showing hematoma, with an air-fluid level, in the right lung, surrounded by an extensive area of pulmonary contusion. Note the small area of ground-glass attenuation in the posterior portion of the contralateral lung, probably also representing contusion. Metallic firearm projectile fragments can be seen in the right scapular region, as can extensive soft-tissue hematoma in the ipsilateral thoracic wall, with fracture of the scapula and costal arches.

Pulmonary lesions were divided among contusions, atelectasis, lacerations, and hematomas. Pulmonary contusion was the most common lesion, being observed in 39 cases (97.5%), presenting as consolidation, ground-glass attenuation, or a combination of the 2. In 5 patients (12.5%) the lesion had a tubular configuration (Figure 1). Pulmonary atelectasis was the second most common lung alteration, being seen in 8 cases (20%), all of which also presented with hemothorax or pneumothorax. Pulmonary laceration (Figure 2) and pulmonary hematoma (Figure 3) were observed in 1 case (2.5%) each. Pulmonary lesions were observed in isolation in 3 cases (7.5%) and in conjunction with other lesions in 37 (92.5%).

### Pleura

**Figure 4:**
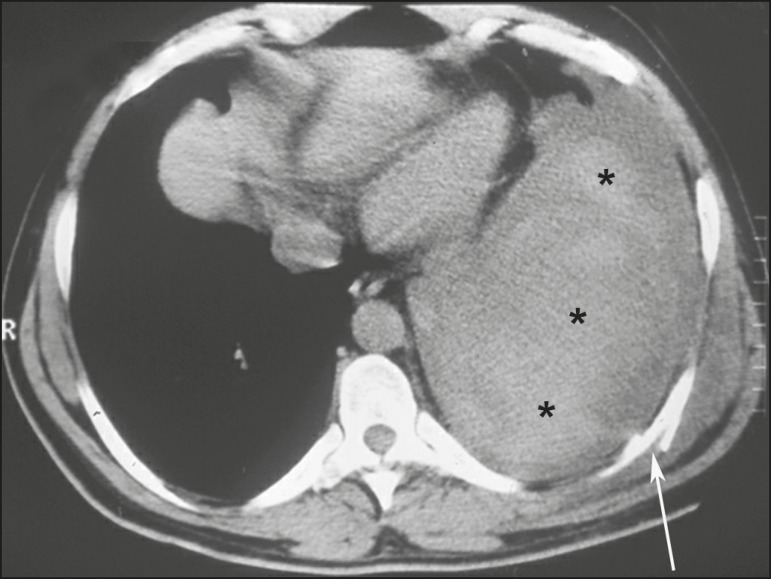
A 38-year-old male victim of a knife attack. Axial CT scan of the chest showing massive pleural effusion with high density and a heterogeneous appearance on the left, consistent with hemothorax (asterisks). Note the misaligned rib fracture (arrow) and hematoma in the corresponding chest wall.

**Figure 5:**
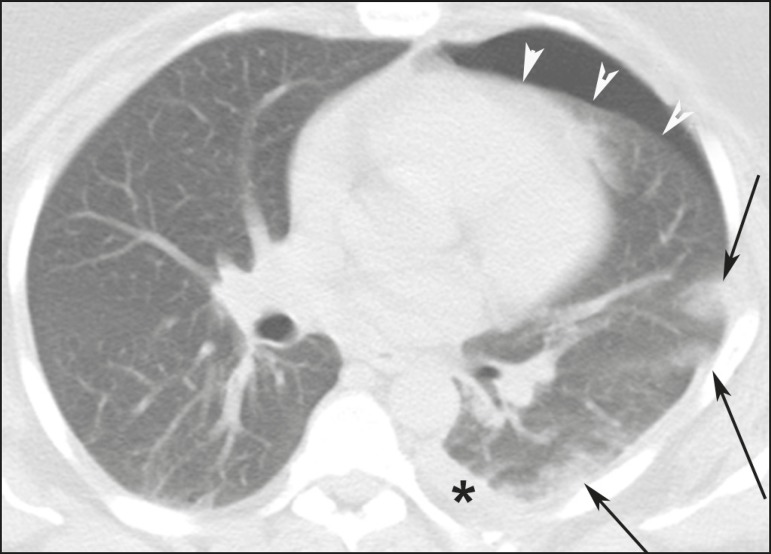
A 22-year-old male gunshot victim. Axial CT scan of the chest showing left anterior pneumothorax (arrowheads), with foci of contusion at the periphery of the ipsilateral lung (arrows) and small pleural effusion (asterisk).

Hemothorax was the second most common tomographic finding, most often seen in the pleural space, and was identified in 31 cases (77.5%), being characterized by pleural effusion of variable density, generally greater than 50 Hounsfield Units (HU) (Figure 4). Hemothorax was accompanied by pulmonary contusion in 30 cases (75%) and by pneumothorax in 18 (45%). Pneumothorax was present in 22 cases (55%) and was accompanied by pulmonary contusion in all of those cases (Figure 5).

### Mediastinum

**Figure 6:**
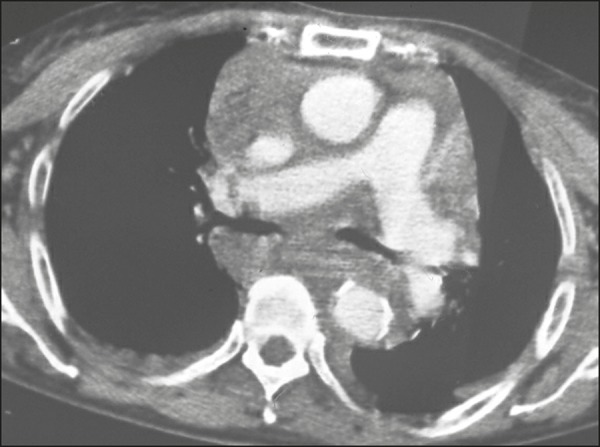
A 21-year-old female gunshot victim. Axial CT scan of the chest after intravenous administration of iodinated contrast media showing mediastinal widening and infiltration by high density material with a heterogeneous appearance, representing mediastinal hemorrhage.

Mediastinal changes were observed in 8 patients, the most common lesions being mediastinal hemorrhage and hemopericardium, each of which was seen in 3 cases (7.5%). Pneumomediastinum was accompanied by pneumothorax in 2 cases. Mediastinal hemorrhage manifested as infiltration of the mediastinal fat by dense material that permeated the mediastinal spaces (Figure 6). Hemopericardium was observed in 3 patients (7.5%) and was characterized by dense material or liquid surrounding the heart. In all cases, the mediastinal lesions were accompanied by lesions at other thoracic sites.

### Diaphragm

**Figure 7:**
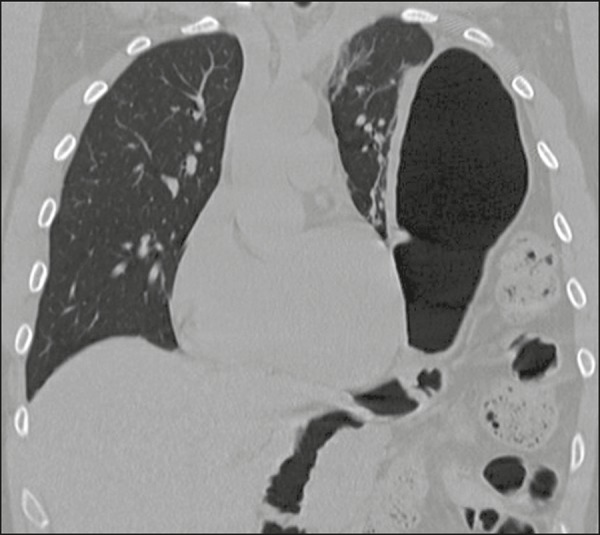
Coronal CT reconstruction of the thorax showing traumatic diaphragmatic hernia on the left, with insinuation of the abdominal contents into the thoracic compartment, including the stomach and intestines.

Diaphragmatic lesions were identified in the left hemidiaphragm in 2 (5%) of the 40 patients evaluated. Lesions of the left hemidiaphragm manifested as intrathoracic herniation of the abdominal viscera, mainly of the stomach (Figure 7).

## DISCUSSION

The American College of Surgeons Committee on Trauma defines trauma as bodily injury characterized by structural changes or physiological imbalance resulting from acute exposure to the transfer of mechanical, thermal, electrical, chemical, or radiative energy^(2)^. Thoracic trauma is traditionally classified as blunt or penetrating. In blunt thoracic trauma, the integrity of the rib cage is maintained, with no loss of continuity or skin break in the area of impact, whereas penetrating thoracic trauma involves the loss of thoracic integrity, with consequent communication between the external environment and the internal contents of the body^(2)^.

Penetrating trauma is usually abrupt and results from the direct application of mechanical force over a small area on the surface of the thorax, resulting in a break in the continuity of the skin and chest wall, usually from a sharp weapon or projectile^(1)^. The resulting tissue injury is produced by laceration of the structures in the path of the projectile or weapon, and its severity depends on the vulnerability of the organs, the speed of the impact, and the fragmentation of the projectile^(1)^. Because weapons other than firearms (knives, etc.) have low energy, the lesions resulting from trauma caused by such weapons are usually restricted to their path. In contrast, firearm projectiles (i.e., bullets) can be of low or high energy, resulting in tissue laceration and causing significant damage, not only along their path but also in adjacent structures^(2,9)^. In our sample, penetrating thoracic trauma by firearm accounted for most (85.8%) of the cases. Wilson et al.^(3)^ found an even more marked predominance in mortality data (gunshot wounds accounting for 99% of deaths from penetrating thoracic trauma). In the present study, the majority (82.4%) of the patients were male, similar to the proportion reported by Wilson et al.^(3)^.

The mediastinal structures (heart, large blood vessels, and esophagus) are highly susceptible to penetrating traumas^(1)^. However, in one prospective study, Renz et al.^(10)^ evaluated 68 gunshot victims with penetrating mediastinal injuries and found that 20 were stable and did not even require surgery. Cases in which there is massive hemothorax, hemopericardium, hemoptysis, mediastinal widening, or marked hypotension call for immediate surgery^(11)^. The majority of gunshot victims with mediastinal injury evolve to shock and cardiac tamponade^(12)^. According to Stassen et al.^(11)^, patients with penetrating trauma caused by a firearm, even if clinically stable, should be referred for surgical treatment. 

### Pulmonary lesions

The CT patterns of pulmonary contusions seen in our study were similar to those reported in the literature, where such contusions are characterized as unilateral or bilateral areas of ground-glass attenuation or consolidation, with a sparse or diffuse distribution that is typically peripheral and nonsegmental^(6-8,13-15)^. In the present study, we observed consolidations in 20 cases (50%), areas of ground-glass attenuation in 18 (45%), and the combination of the two in 14 (35%). The finding of a tubular wound, which is quite specific for pathway injury in penetrating trauma, was observed in only 5 cases (12.5%). Atelectasis represents secondary volumetric reduction of the lung or part of it, being characterized by localized opacities, corresponding to subsegmental atelectasis, and opacities in the posterior portions of the lungs, caused by pleural effusion or pneumothorax^(2,13)^. Blunt or penetrating trauma can cause pulmonary lacerations, which tend to resolve within 3-5 months^(6)^ but can evolve to pneumatocele formation and potential complications such as abscess and bronchopleural fistula. In the present study, we observed pulmonary laceration in only one case, which was a case of penetrating thoracic trauma. That pulmonary laceration was characterized by consolidation containing air, with the same imaging aspect reported in the literature^(2)^. Blunt or penetrating trauma can also cause pulmonary hematomas^(2)^, which are blood-filled parenchymal lesions, characterized on imaging by lesions with an air-fluid level or by soft-tissue opacities^(2)^. In our study, which considered only acute thoracic trauma, we observed pulmonary hematoma in only 1 case (2.5%). The low prevalence of pulmonary hematoma in our sample is probably attributable to the fact that this type of lesion is typically detected later in the evolution of the injury, a period that was not evaluated in the presented study.

Injuries along the trajectory of projectiles and knives are characterized on CT by images indicative of air, blood, bone, or metal fragments. In general, projectiles cause lesions that are more extensive, and CT has a great capacity to delineate the pathway of a projectile, as well as to evaluate the risk of mediastinal lesions. Analysis via lung, mediastinum, and bone windows is essential for better characterization of the damage^(9)^.

### Pleural lesions

In our sample, hemothorax occurred in 77.5% of the cases, a frequency higher than the 32% reported by Karaaslan et al.^(15)^. In our study, hemothorax occurred in conjunction with pulmonary contusions in 75% of cases and in conjunction with pneumothorax in 45% of cases. This combination of lesions has previously been reported^(2)^. Hemothorax can be caused by pulmonary contusions/lacerations or pleural lesion, as well as by injury to the mammary/intercostal arteries, heart, or large blood vessels^(2)^. In our study, we observed a wide range of blood density values in the pleural cavity. A variety of blood density values were also described by Karaaslan et al.^(15)^, ranging from that of water (0 HU) up to 80 HU. Pneumothorax, manifesting as air in the pleural cavity, was detected in 22 cases (55%), representing the second most common pleural alteration, in our sample. Wagner et al.^(16)^ found pneumothorax to be the second most common intrathoracic lesion in cases of thoracic trauma. In the present study, the combination of rib fractures and pneumothorax was seen in 12 cases (30%). Karaaslan et al.^(15)^ observed the same combination in 25% of their cases.

### Mediastinal lesions

Traumatic mediastinal lesions result from severe trauma and are typically associated with high mortality. In our study, mediastinal lesions were accompanied by pulmonary or pleural lesions in all cases. That association has also been reported in the literature^(6,3,15,17)^. Hemopericardium and mediastinal hemorrhage were seen in three (7.5%) of our patients, an incidence similar to the 10% observed by Shanmuganathan et al.^(9)^. In the present study, vascular lesions were not identified on any of the CT scans evaluated, which was quite likely due to the severity of the trauma in patients who have vascular lesions, especially lesions of the aorta, as well as to the difficulties of accessing the sites of the traumatic events, patient transport, and low hospital survival rates. Mediastinal hemorrhage manifests as blurring of the mediastinal fat image, with accumulation of dense material in the interior, or as a dense expansile lesion (hematoma) and can be caused by bleeding from small arterial or venous vessels, or even from extensive aortic lesions^(5,9)^. The presence of mediastinal hemorrhage suggests vascular lesion, necessitating a study with venous contrast injected by an infusion pump^(9)^. In three (7.5%) of the cases evaluated in our study, hemopericardium was accompanied by penetrating trauma, a combination that has also been reported in the literature^(2)^. Hemopericardium manifests as blood in the pericardial sac, which appears as a collection of fluid of varying density surrounding the heart on CT. Clinically, hemopericardium can be associated with cardiac tamponade^(2)^. In our sample, all of the cases of hemopericardium were accompanied by other pulmonary or pleural injuries, a combination reported by Restrepo et al.^(12)^, who also cited traumatic lesions in the heart, coronary artery, and aorta, as well as at other thoracic sites^(9)^. Mediastinal and periaortic hematomas were observed in all cases of aortic lesion in our study. Dyer et al.^(18)^ classified such hematomas as important signs for the diagnosis of aortic lesion, assigning them a negative predictive value of nearly 100%. The absence of tracheobronchial lesions in our sample was probably due to their extreme rarity.

### Diaphragmatic lesions

Diaphragmatic lesions were observed by CT in two cases (5%), which is within the 0.8-8% range reported in the literature^(19,20)^. In both of our cases, the lesion was in the left hemidiaphragm, with gastric herniation. Herniation of the stomach or colon into the thorax is the finding that is most characteristic of rupture of the left hemidiaphragm^(4,19,20)^. Because CT allows rapid, efficient diagnosis of diaphragmatic lesions, it facilitates early treatment, avoiding the need for late repair, which, due to the presence of fibrous tissue, can be more difficult^(18)^. Coronal and sagittal reconstructions increase the diagnostic accuracy of the method^(4,19,20)^. The co-occurrence of abdominal lesions and hemidiaphragm rupture has been reported in the literature^(4,19)^. In our sample, we observed diaphragmatic rupture accompanied by hemoperitoneum in two cases and by splenic injury in one.

The main limitations of our study are that it was a retrospective study, that it included a relatively small number of cases, that it did not contemplate the clinical evolution of the patients studied, and that no control CT examinations were performed. In conclusion, this study demonstrated that, in cases of penetrating thoracic trauma, chest CT can characterize the affected organs, as well as determining the trajectory, severity, and extent of the lesions, representing a useful tool in estimating mortality risk and playing a crucial role in the decision-making process related to the choice of therapeutic approaches.
